# Cerebral and Peripheral Hemodynamics Across Wakefulness and NREM Sleep

**DOI:** 10.1111/jsr.70180

**Published:** 2025-08-20

**Authors:** Vidhya V. Nair, Brianna R. Kish, Hideyuki Oshima, Qiuting Wen, Yunjie Tong, A. J. Schwichtenberg

**Affiliations:** ^1^ Weldon School of Biomedical Engineering, Purdue University West Lafayette Indiana USA; ^2^ Graduate School of Engineering and Science, Shibaura Institute of Technology Tokyo Japan; ^3^ Department of Radiology and Imaging Sciences, Indiana University School of Medicine Indianapolis Indiana USA; ^4^ Department of Human Development and Family Science College of Health and Human Sciences, Purdue University West Lafayette Indiana USA

**Keywords:** cerebral hemodynamics, low‐frequency oscillations, non‐rapid eye movement sleep, peripheral hemodynamics

## Abstract

Wake/sleep‐related changes in cerebral hemodynamic oscillations are well established, but similar changes in peripheral hemodynamics remain largely understudied. Moreover, how the relationship between cerebral and peripheral hemodynamics varies across sleep–wake states is not well understood, despite evidence that these oscillations in the low‐frequency range are strongly coupled during wakefulness. In this study, we investigated the temporal and spectral characteristics of cerebral and peripheral hemodynamics, as well as their low‐frequency coupling, across sleep and wake states. To this end, we simultaneously measured cerebral hemodynamics using functional magnetic resonance imaging (fMRI) of the brain and peripheral hemodynamics using near‐infrared spectroscopy (NIRS) of the fingertips in 10 healthy participants (6 females; age 19–24 years, mean ± SD: 20.90 ± 1.59 years) during wakefulness and non‐rapid eye movement (NREM) sleep. Our results show that during sleep, cerebral hemodynamics differ markedly from peripheral hemodynamics in both oscillation amplitude and spectral power. Furthermore, low‐frequency coupling between cerebral and peripheral hemodynamics becomes desynchronized during NREM3 sleep. These findings support the notion that NREM3 sleep plays a key role in the optimal restoration of cerebral vasomotion.

## 1 Introduction

Sleep is a fundamental, reversible neurobehavioral state that promotes physiological restoration and optimal brain function through coordinated changes at the cellular, circuit, and systems levels (Buysse 2014). In mammals, sleep comprises rapid eye movement (REM) and non‐REM (NREM) stages; NREM sleep is further divided into light sleep (NREM1 and NREM2) and deep slow‐wave sleep (NREM3). Individuals cycle through these stages in patterns characterised by distinct neuronal, muscular, respiratory, and vascular activity (Carley and Farabi 2016; Saper et al. 2010).

Cerebral hemodynamic changes during sleep have been extensively characterised using functional magnetic resonance imaging (fMRI). Previous studies demonstrate that global cerebrovascular signal fluctuations increase during light NREM sleep (Fukunaga et al. 2006; Horovitz et al. 2008; Vijayakrishnan Nair et al. 2023) and document that functional connectivity undergoes significant reorganisation (Boly et al. 2012; Larson‐Prior et al. 2009). Recent findings also reveal that spatio‐spectral variations in cerebrovascular signals correspond to dynamic cortical states during NREM sleep (Song et al. 2022).

In contrast, peripheral hemodynamic changes across sleep–wake states and their relationship to cerebral hemodynamics remain comparatively understudied. To date, most research on peripheral hemodynamics focuses on extracting autonomic indicators—such as heart rate, respiratory rate and variability—from photoplethysmography to assist with sleep stage scoring (Altini and Kinnunen 2021; Birrer et al. 2024; Imtiaz 2021), detection of sleep disordered breathing (Grote et al. 2003; Massie et al. 2023; Sommermeyer et al. 2012; Vulcan et al. 2021), or monitoring blood pressure and vascular elasticity in wearable devices (Yilmaz et al. 2023). However, peripheral hemodynamic oscillations, particularly in the low‐frequency range (0.01–0.1 Hz), also reflect non‐neuronal systemic physiological processes that may be distinct from autonomic signals (Hocke et al. 2016). Notably, these systemic oscillations measured at the periphery using near‐infrared spectroscopy (NIRS) correlate with cerebral hemodynamics recorded via fMRI during wakefulness (Tong et al. 2011, 2012) and light NREM sleep (Vijayakrishnan Nair et al. 2023). These findings suggest that systemic physiological processes may contribute to variability in cerebral hemodynamic signals across sleep and wake states.

Characterising the coupling between peripheral and cerebral hemodynamics during sleep has important implications. Fluctuations in cerebral blood volume help drive cerebrospinal fluid movement within the glymphatic system, which is more active during sleep and plays a key role in waste clearance (Fultz et al. 2019; Nair et al. 2025; Xie et al. 2013; Yang et al. 2022). Understanding how peripheral hemodynamics interact with cerebral hemodynamics could provide new insights into the role of systemic physiology in neurofluid dynamics and related disorders such as Alzheimer's disease and hydrocephalus. Moreover, quantifying this coupling across sleep–wake states could inform novel, non‐invasive approaches for sleep monitoring beyond conventional polysomnography and potentially identify vascular anomalies linked to sleep‐related conditions (Chong et al. 2022).

In our previous work, we demonstrated strong correlations between peripheral blood signals measured at the fingertip using NIRS and cerebral signals captured by fMRI (Tong et al. 2012). Building on this foundation, the present study investigates how this brain–body hemodynamic coupling varies across sleep and wakefulness. Using a multimodal approach combining fMRI, NIRS and EEG, we simultaneously recorded cerebral hemodynamics, peripheral hemodynamics, and electrical brain activity. Specifically, we aim (1) to characterise changes in amplitude fluctuations and frequency content of cerebral and peripheral hemodynamics across sleep–wake states, and (2) to assess low‐frequency coupling between these signals during sleep and wakefulness. By clarifying this underexplored interaction, our findings seek to advance the understanding of sleep's effects on systemic and cerebral vascular physiology.

## 2 Methods

### 2.1 Participants

This study was approved by Purdue University's Institutional Review Board (IRB‐2020‐1329) and included 10 healthy participants (6 females) aged 19–24 (20.90 ± 1.59) years. Written informed consent was obtained from all participants before enrollment. Actigraphy was used to track each participant's sleep–wake cycles for at least 4 days before the experiment to ensure they adhered to their usual sleep schedules. Additionally, to allow for sound acclimation, they were instructed to listen to sound recordings from the MR scanner at bedtime for two to three nights before their scheduled bedtime scan. To capitalise on elevated homeostatic and circadian sleep pressures, participants were asked to wake up 3 h earlier than usual the morning of their scan, and the scan sessions began roughly 1 h after their usual sleep onset time. Participants were asked to relax and attempt to fall asleep in the scanner. Most participants slept for one sleep cycle (*n* = 6), but a few did not achieve deep sleep (*n* = 4). However, everyone achieved light sleep (NREM1, NREM2) or deep sleep (NREM3). For a breakdown of data across participants, see Table S1.

### 2.2 Data Acquisition

**FIGURE 1:**
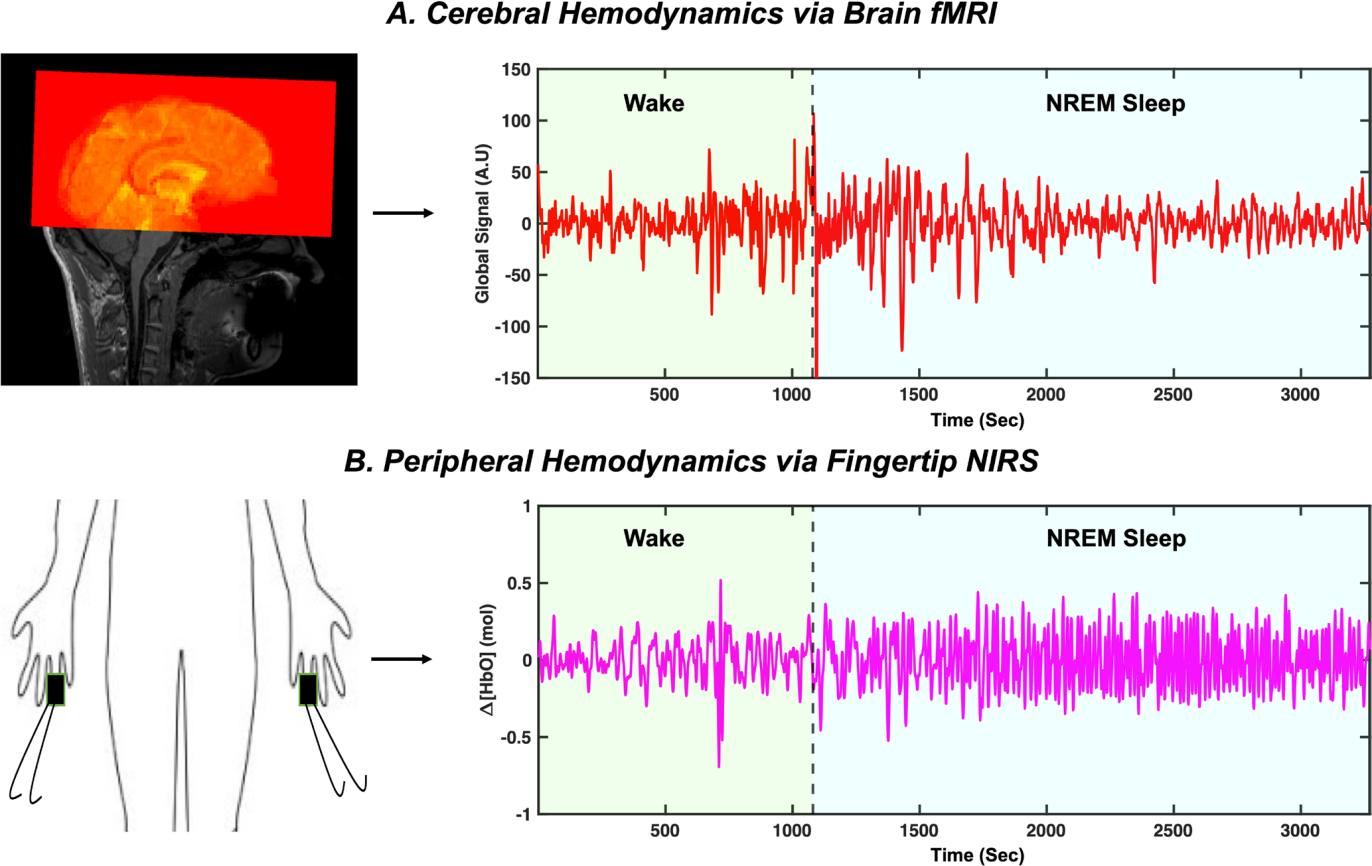
Overview of the study design illustrating (A) fMRI scan positioning of brain to capture global cerebral hemodynamics and (B) placement of NIRS optodes on the fingertips to capture peripheral hemodynamics together. Δ[HbO]—change in concentration of oxyhemoglobin; A.U—arbitrary units; fMRI—functional magnetic resonance imaging; NIRS—near‐infrared spectroscopy; NREM—non‐rapid eye movement sleep.

An overview of the study design and data acquisition is described in Figure 1. All MRI data were acquired using a 3 T SIEMENS MRI scanner (Magnetom Prisma, Siemens Medical Solutions, Erlangen, Germany) with a 64‐channel head coil. The MR scans included structural T1‐weighted MPRAGE (TR/TE: 2300/2.26 ms, 192 slices per slab, flip angle: 8°, resolution: 1.0 × 1.0 × 1.0 mm) and fMRI (FOV = 230 mm, acquisition matrix = 92 × 92, 48 slices, voxel size = 2.5 × 2.5 × 2.5 mm, TR/TE = 1440/30.6 ms, echo‐spacing = 0.51 ms, flip angle = 35°, multiband acceleration factor = 8, multi‐slice mode: interleaved) of the brain (Figure 1A). Peripheral hemodynamics were recorded (Figure 1B) at a sampling frequency of 31.25 Hz from two fingertips (fourth finger of both arms) using a Continuous Wave (CW) NIRS system (NIRScoutXP NIRx Medizintechnik GmbH, Berlin, Germany) with two laser sources, each combining two wavelengths (785 and 830 nm) and MRI‐compatible NIRS probes with 10 m long optical fibres. For sleep scoring, a 32‐channel Electroencephalography (EEG) was also simultaneously acquired using an MRI‐compatible EEG system (BrainAmp MR, Brain Products GmbH, Gilching, Germany). All recorded physiological signals were synchronised in time. EEG recording software automatically received markers from the MR system when the fMRI scan started. For NIRS, a marker was manually inserted by the operator at the same time as the start of the fMRI scan.

### 2.3 Data Preprocessing

#### 2.3.1 EEG Preprocessing and Sleep Scoring

The preprocessing of EEG signals included two steps: First, the MR gradient artefacts and cardioballistic artefacts were removed from the EEG signals using the Brain Vision Analyser software (Brain Vision, Morrisville, USA). Second, the ocular, muscular or electrocardiographic artefacts were removed using independent component analysis on a participant‐by‐participant basis, using EEGLAB v2021.0 toolbox (Delorme and Makeig 2004). Following that, the data were scored into sleep states (NREM1, NREM2, NREM3) in 30‐s periods, using the American Academy of Sleep Medicine scoring manual criteria (Berry et al. 2012). Sleep staging was completed by a graduate student (HO) under the supervision of the study Co‐PI (AJS). Given the noisy nature of the MR environment, the collected data contained more artefacts than a typical polysomnography (i.e., more epochs of indeterminate sleep than a typical recording). For analyses, only epochs with clear awake, NREM1, NREM2 or NREM3 classifications were retained (checked by a master coder with over 10 years of experience). The analysis included sufficient durations of wakefulness (93 min), NREM1 (48 min), NREM2 (63 min) and NREM3 (66 min). Given the nature of the utilised protocol (high sleep inertia and only one cycle into deep sleep), no REM sleep was scored or used in the analyses.

#### 2.3.2 fMRI And NIRS Preprocessing

fMRI data were preprocessed using FSL (FMRIB Expert Analysis Tool, v6.01; Oxford University, UK (Jenkinson et al. 2012)) and MATLAB (MATLAB 2021b, The MathWorks Inc. Natick, MA, 2000). The preprocessing pipeline included motion correction (*FSL MCFLIRT*), slice‐timing correction (*FSL SLICETIMER*), and registration to the structural space. The global mean signal (GS) was then extracted across the entire brain (Figure 1A). The raw NIRS data were converted to Δ[HbO] time series (Figure 1B) using the nirsLAB analysis package (v2019.4, NIRx Medical Technologies LLC.; Los Angeles) (Xu et al. 2014), based on the modified Beer– Lambert law. Both GS and Δ[HbO] time courses were linearly detrended and normalised by their mean value (Korponay et al. 2024) before all further analysis to account for the differences in signal baseline between the segments. The Δ[HbO] signals from both fingertips exhibited high correlations (0.77 ± 0.12). Therefore, these signals from two fingertip channels were averaged for all participants (except two participants, where one channel each was not usable since cardiac pulsations were not sufficiently detected) to increase the signal‐to‐noise ratio.

### 2.4 Data Analysis

All analyses in this study were performed after identifying continuous uninterrupted low‐motion segments of wake/NREM sleep states with a minimum of 3 continuous minutes.

#### 2.4.1 Amplitude Variation Analysis

This analysis was employed to understand the temporal changes in amplitude fluctuations of both GS and Δ[HbO] signals between wake and sleep states. This was quantified by calculating the variance across continuous 3‐min segments of wakefulness, NREM1, NREM2 and NREM3 (as data were available).

#### 2.4.2 Spectral Power Variation Analysis

The changes in spectral frequency content of GS and Δ[HbO] signals were assessed across continuous 3‐min segments of wakefulness, NREM1, NREM2 and NREM3 for each participant. Welch's power spectral density estimate (MATLAB ‘pwelch’) was used to calculate the power spectra, with the default Hamming window of segment length and no overlap. Further, the average spectral power estimates were also calculated using MATLAB ‘bandpower’ in the LFO range for every behavioural state.

#### 2.4.3 Coupling Strength Analysis

The GS and Δ[HbO] signals were first bandpass filtered to the LFO range of 0.01–0.1 Hz, using a zero delay fourth‐order Butterworth filter to extract the corresponding LFOs (LFO_GS_ and LFO_NIRS_). Then, the coupling strength between them was determined by calculating maximum cross‐correlation coefficients (MCCCs) and corresponding time delays (MATLAB xcorr, maximum lag range: ±15 s) between LFO_GS_ and LFO_NIRS_. The absolute maximum value from the calculated CCCs was identified as the MCCC with its original arithmetic sign. Based on previous research, only the MCCCs above the statistically established threshold of 0.3 (*p*‐value < 0.01 for positive MCCCs) or below −0.3 (*p*‐value < 0.01 for negative MCCCs) were considered significant in the LFO range (Hocke et al. 2016; Yao et al. 2019).

#### 2.4.4 Statistical Analysis

Non‐parametric Skillings–Mack tests (Skillings and Mack 1981) were used to test for statistical significance in the assessed parameters between the behavioural states (wake, NREM1, NREM2 and NREM3), given the non‐independence of observations and non‐normal distribution (normality determined via one‐sample Kolmogorov–Smirnov test). Additionally, the non‐parametric effect size (Cliff's *d*) (Vargha and Delaney 2000) of these observations is also reported. Skillings–Mack test comparing four conditions (i.e., wake, NREM1, NREM2, NREM3) at a 5% significance level yields a statistical power of ~0.73–0.95, with the sample size of *N* = 10.

## 3 Results

### 3.1 Hemodynamic‐Oscillation Amplitudes Significantly Change Only in the Brain During NREM Sleep

**FIGURE 2:**
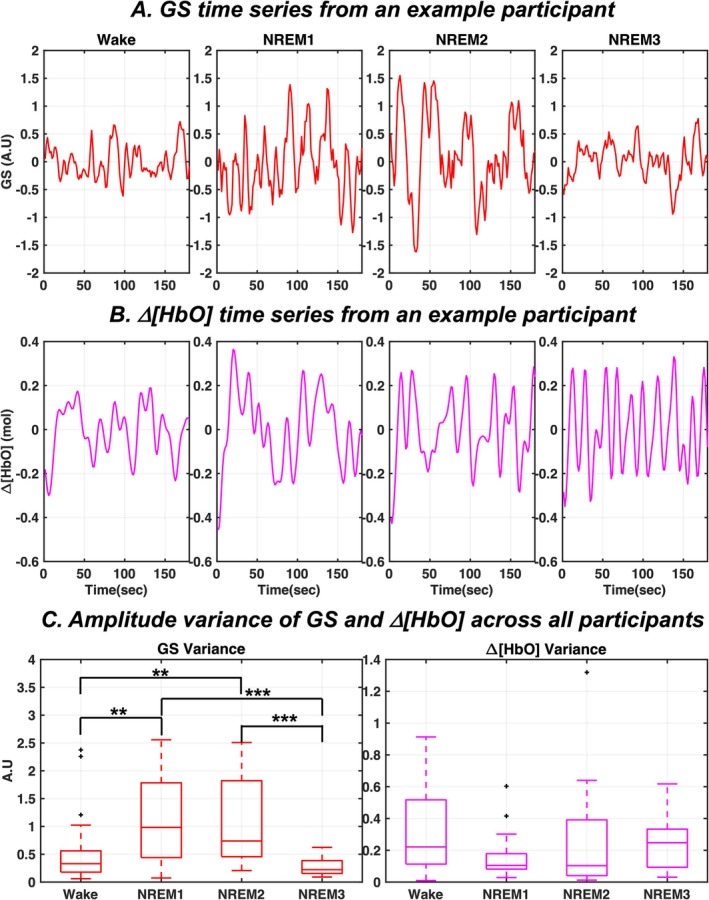
Amplitude variation in GS and △[HbO] signals across wake and NREM sleep states. (A) Continuous 3‐minute segments of each behavioural state from an example participant illustrating the variations in fluctuation amplitude. (B) Group results capturing the differences in variance in GS and △[HbO] oscillations between the wake and NREM sleep states for all participants. Δ[HbO]—change in concentration of oxyhemoglobin; A.U—arbitrary units; GS—global signal; NREM—non‐rapid eye movement sleep; *p‐value < 0.05; **p‐value < 0.01; ***p‐value < 0.001.

The time series plots of GS and Δ[HbO] signals from a representative participant are provided in Figure 2A,B, respectively. As illustrated, the amplitude fluctuations of hemodynamics increase only in the brain GS during light NREM sleep states (NREM1 and NREM2) compared to wakefulness. During the deep NREM3 state, the fluctuation amplitude of GS lowers and resembles wakefulness patterns. Group results reveal the same pattern with statistically significant changes in amplitude fluctuations quantified by signal variance across wake and NREM sleep states (Figure 2C, Left Panel). In detail, the GS variance during light NREM sleep is significantly higher (*p*‐value = 0.0035 for NREM1 and *p*‐value = 0.0019 for NREM2) compared to resting wakefulness, with large effect sizes (Cliff's *d* = 0.5369 for NREM1 and Cliff's *d* = 0.5793 for NREM2). Conversely, the Δ[HbO] signals from the peripheries did not show any notable variations in fluctuation amplitude between wake and NREM sleep states, as illustrated in the example time courses from a representative participant (Figure 2B) and group results quantified by Δ[HbO] signal variance (Figure 2C, Right Panel).

### 3.2 Spectral Power in LFO Band Changes Only in the Brain During NREM Sleep

**FIGURE 3:**
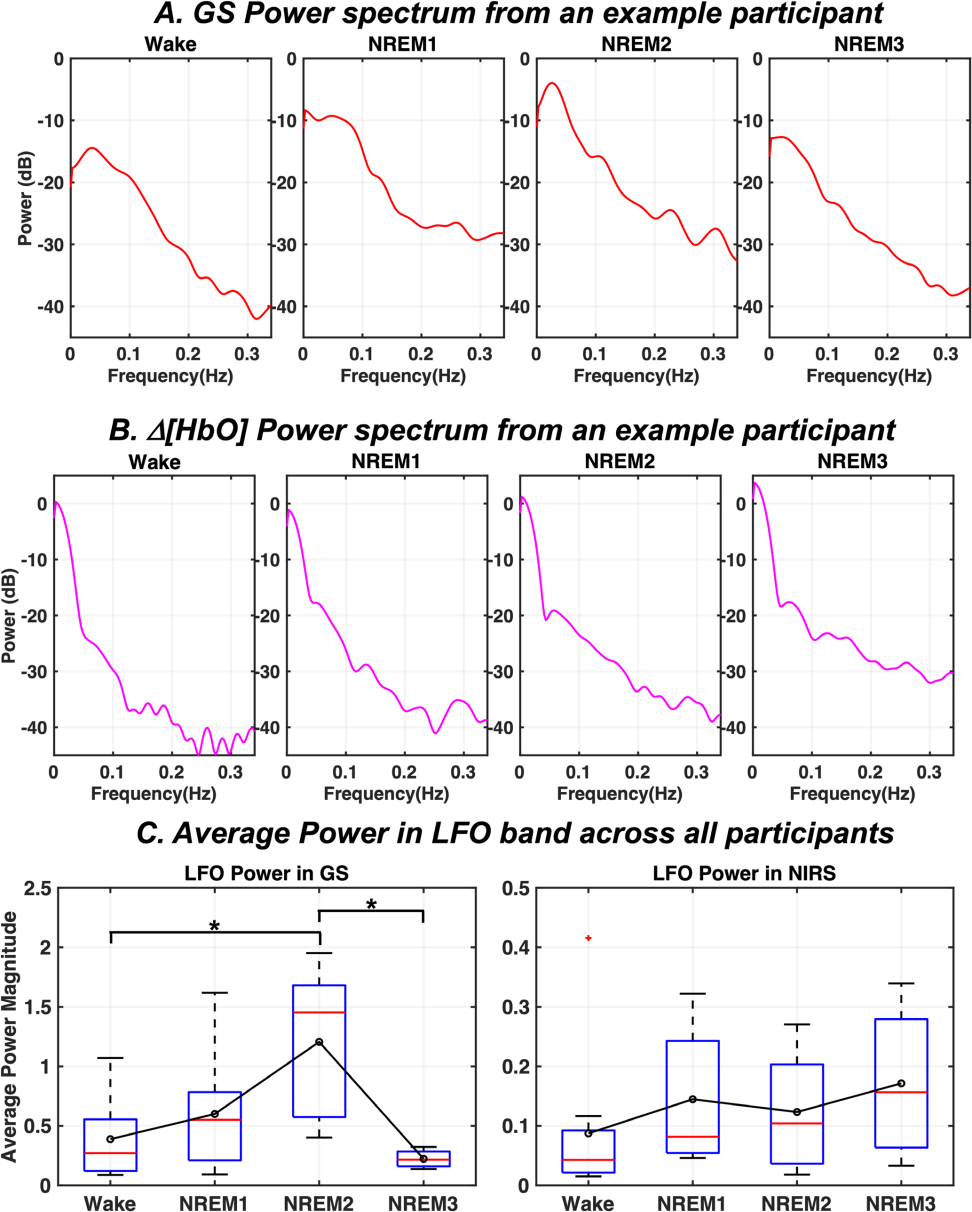
Spectral power variations in GS and △[HbO] signals across wake and NREM sleep states. Power spectra of continuous 3‐minute segments of (A) GS and (B) △[HbO] for each behavioural state from an example participant. (C) The average power estimates of GS and △[HbO] signals in the LFO band for all participants. In the box plots, the median and mean of the data are respectively indicated by the red horizontal line and the small black circle. Δ[HbO]—change in concentration of oxyhemoglobin; GS—global signal; LFO—low frequency oscillations; NREM—non‐rapid eye movement sleep; *p‐value < 0.05; **p‐value < 0.01.

Figure 3A,B, respectively, illustrate the power spectra from continuous 3‐min segments of GS and Δ[HbO] signals from a representative participant for each behavioural state. The spectral power in GS increases during light sleep compared to wakefulness, and decreases during deep NREM3 sleep (Figure 3A). Spectral power of Δ[HbO], however, does not show such changes (Figure 3B). It can also be seen that LFO dominates the spectrum in GS and Δ[HbO] signals across all behavioural states. Moreover, the changes in spectral power in GS during NREM sleep states are more evident in the LFO range. Across all participants, compared to resting wake state, LFO power in GS is relatively higher with large effect sizes during NREM1 (*p*‐value = 0.7685, Cliff's *d* = 0.4778) and significantly higher during NREM2 (*p*‐value = 0.0175, Cliff's *d* = 0.7333). However, NREM3 shows a significant drop in LFO power with a large effect size in GS (*p*‐value = 0.0158, Cliff's *d* = 1) compared to light sleep (Figure 3C, Left Panel). Group results of average LFO power variations in Δ[HbO] between wake and NREM sleep states reveal no such patterns (Figure 3C, Right Panel) with low effect sizes between states (Cliff's *d* < 0.2 for all comparisons).

### 3.3 Cerebral and Peripheral LFOs Show Significant Positive Correlations During all States Except NREM3

**FIGURE 4:**
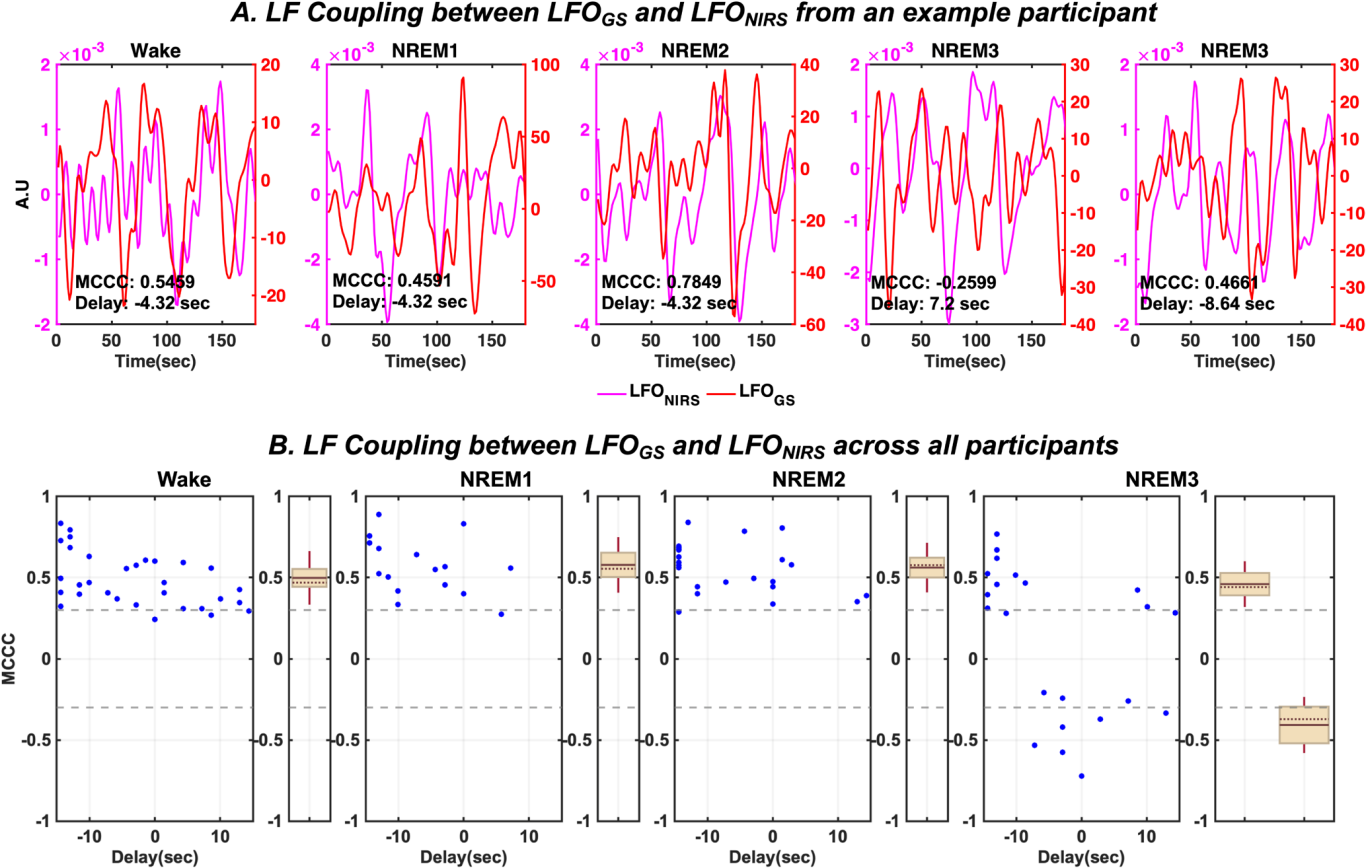
Strong coupling exists between LFO_GS_ LFO_NIRS_ across all behavioural states except NREM3. Results of MCCCs and corresponding delays between LFO_GS_ and LFO_NIRS_ during continuous 3‐minute segments of wakefulness, NREM1, NREM2 and NREM3 for (A) a representative participant and (B) for all participants enrolled in the study. In panel B, the blue dots represent the MCCCs for all 3‐minute segments for each behavioural state, brown solid line represents the mean, brown dotted line represents the median, brown whiskers represent one standard deviation of the MCCCs and the grey dashed line represent the threshold of statistical significance for MCCCs in the LFO range. Δ[HbO]—change in concentration of oxyhemoglobin; A.U—arbitrary units; GS—global signal; LFO—low frequency oscillations; MCCC—maximum cross‐correlation coefficient; NIRS—near‐infrared spectroscopy; NREM—non‐rapid eye movement sleep.

Figure 4A illustrates the coupling LFO_GS_ and LFO_NIRS_ during continuous 3‐min segments of wakefulness, NREM1, NREM2 and NREM3 from a representative participant. It can be inferred that LFO_GS_ exhibit significant positive correlations (MCCC > 0.3) with LFO_NIRS_, during all states except NREM3. NREM3 sleep shows periods of significant positive (MCCC > 0.3) as well as negative (MCCC < −0.3) correlations between LFO_GS_ and LFO_NIRS_.

Across all participants (Figure 4B), significant mean positive correlations (wakefulness: 0.50 ± 0.17; NREM1: 0.58 ± 0.17; NREM2: 0.56 ± 0.15) were seen during wakefulness, NREM1, and NREM2. However, during deep NREM3, MCCCs clustered into positive (0.46 ± 0.14) and negative (−0.41 ± 0.17) groups. As for the time delays, it can be seen that there is high variability during all behavioural states wakefulness: −3.87 ± 9.47 s, NREM1: −7.34 ± 7.15 s, NREM2: −7.14 ± 9.18 s, NREM3: −8.73 ± 9.94 s for positive MCCCs and 0.16 ± 6.49 s for negative MCCCs. Negative delay indicates that LFO_GS_ leads LFO_NIRS_. Moreover, it can also be seen that the delay times tend to cluster into negative values (i.e., LFO_GS_ leading LFO_NIRS_) during light NREM sleep states (Figure 4B).

**FIGURE 5:**
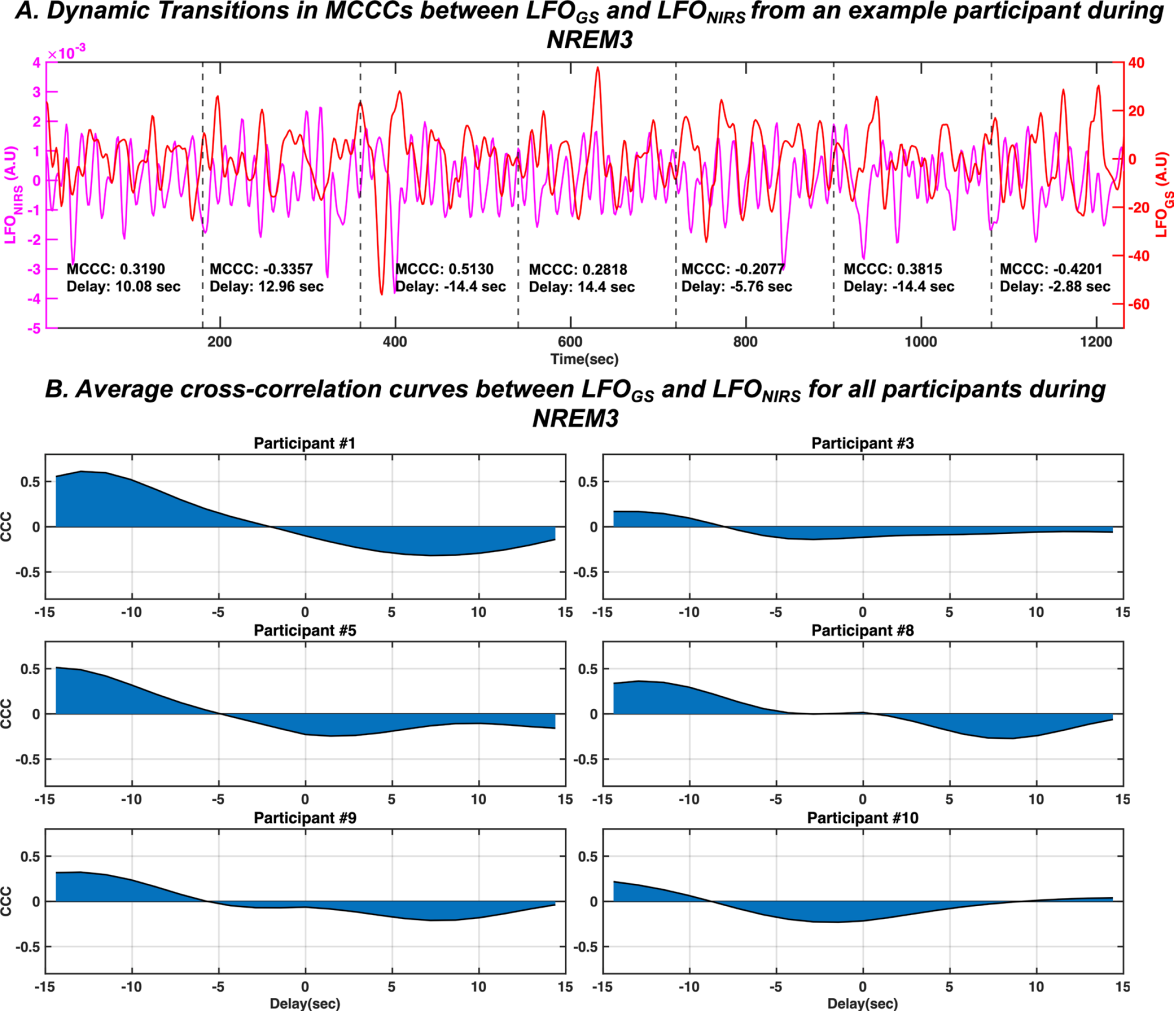
Altered coupling between LFO_GS_ and LFO_NIRS_ during NREM3. (A) Spontaneous dynamic transitions in coupling between LFO_GS_ and LFO_NIRS_ during NREM3 from an example participant. (B) Cross correlation curves between LFO_GS_ and LFO_NIRS_ averaged across all 3‐minute segments of NREM3 state for each participant with NREM3 sleep. CCC—cross‐correlation coefficient; GS—global signal; LFO—low frequency oscillations; NIRS—near‐infrared spectroscopy; NREM—non‐rapid eye movement sleep.

In addition, our analysis also revealed that the MCCCs between LFO_GS_ and LFO_NIRS_ exhibit spontaneous dynamic transitions during NREM3, as illustrated from an example participant's continuous NREM3 data for a duration of 20.8 min in Figure 5A. This implies that there is no consistent pattern as to when these signals are positively/negatively correlated during NREM3 sleep. To further probe into this altered coupling between LFO_GS_ and LFO_NIRS_ during NREM3 sleep, cross‐correlation curves between LFO_GS_ and LFO_NIRS_ averaged across all 3‐min segments of NREM3 state for each participant are plotted in Figure 5B. Each participant, during NREM3, illustrates a positive peak as well as a negative peak of similar magnitudes, indicating the desynchrony between LFOs travelling to peripheries and the brain during NREM3.

## 4 Discussion

Overall findings from the present study are twofold. First, haemodynamics in the brain remarkably differ in both oscillation amplitude and spectral power from peripheral haemodynamics during NREM sleep stages. Second, the LF coupling between cerebral and peripheral haemodynamics becomes desynchronized during NREM3 sleep. The physiological significance of these findings is discussed below.

### 4.1 Fluctuation Amplitude and Spectral Power Changes in Cerebral and Peripheral Hemodynamics From Wake to NREM3

Our data demonstrate that the amplitude fluctuations of cerebral hemodynamics significantly increase during light NREM sleep (NREM1 and NREM2) and then decrease significantly during deep sleep (NREM3). Fluctuation levels of peripheral hemodynamics, however, stay the same from wake to NREM3. To our knowledge, this is the first study to report this discrepancy in hemodynamic fluctuation levels between the brain and periphery during NREM sleep.

The increase in variability of brain hemodynamics during light NREM sleep has been reported before (Fukunaga et al. 2006; Horovitz et al. 2008; Vijayakrishnan Nair et al. 2023). Sleep‐state dependent rise in autonomic neural activity may be considered as a potential contributor (Özbay et al. 2019) towards this increased variability in brain hemodynamics. For instance, hypoventilation during sleep could result in increased levels of blood CO_2_ (Sowho et al. 2014). As a result, the autonomic nervous system would be activated (Agassandian et al. 2003; Fink et al. 2018) to ensure proper blood flow to the brain until breathing patterns stabilise and the eupneic CO_2_ level for sleep is achieved (Dempsey et al. 2004). In the same manner, sleep‐induced reduction in blood pressure (Douma and Gumz 2018) could also lead to large blood flow fluctuations in the cerebral cortex (Hudetz et al. 1992). A potential reason as to why these autonomically controlled large hemodynamic fluctuations do not show up at the fingertips might be due to the differences in density of autonomic innervation between the two locations. The brain's vasculature has an abundant supply of both sympathetic and parasympathetic nerve fibres for the optimal maintenance of cerebral perfusion (Koep et al. 2022), whereas the blood vessels at the fingertips lack parasympathetic innervation, as opposed to only sympathetic innervation (Glatte et al. 2019; Oaklander and Siegel 2005). As a result, the full range of autonomic variability during light sleep might not be captured at the fingertips. The spectral power changes in cerebral and peripheral hemodynamics also reflect the changes in amplitude fluctuations. This is especially noticeable in the LFO range with a gradual rise in LFO power from wake through NREM2 states, solely in the cerebral hemodynamics (Figure 3). Interestingly, our analyses also illustrate that this rise reached statistical significance within the narrow LFO range of 0.02–0.04 Hz, believed to stem from autonomic neural activity (Refer to Figure S2).

NREM3 sleep is a period where the brain signal fluctuation amplitude and the spectral power (particularly in the LF range) drop, potentially due to the decline in reciprocal autonomic neural signalling. Moreover, NREM3 sleep may also have a restorative role, actively reducing the vasomotor activities in the cerebral cortex, which are discussed in detail in section 4.2.

### 4.2 LF Coupling Between Cerebral and Peripheral Hemodynamics From Wake to NREM3

LFOs from the fingertips have been illustrated to be positively correlated with cerebrovascular LFOs during awake (Tong et al. 2012) as well as light NREM sleep (Tuunanen et al. 2024; Vijayakrishnan Nair et al. 2023) states. Intriguingly, this relationship is lost during NREM3 (Figure 4), with the signals spontaneously transitioning between positive and negative correlations across the duration of NREM3 (Figure 5). A consistently high positive correlation between brain and peripheral LFOs suggests that non‐neuronal systemic physiology accounts for a considerable part of the cerebral haemodynamic variability observed during wakefulness in our previous studies (Tong et al. 2012; Tong and Frederick 2010). Additionally, the consistent delays (~3–7 s) between brain and peripheral LFOs indicate an endogenous cardiopulmonary source behind these LFO signals, suggesting that they are physiological rather than neuronal in origin (Tong et al. 2012).

In the present study, we observed a similar high positive correlation between brain and peripheral LFOs in the NREM1 and NREM2 sleep states. However, this correlation was not present during NREM3. This finding is consistent with a prior study that reported a reduction in vasomotion specifically in the brain, compared to the body, during slow‐wave (NREM3) sleep (Zhang and Khatami 2014). These findings therefore suggest a unique physiological decoupling between cerebral and peripheral vascular LFOs exclusively during NREM3 sleep.

Based on these findings, it may be possible that the brain generates a large, synchronised intrinsic activity, distinct from the non‐neuronal systemic LFOs (originating from the cardiopulmonary system that typically dominate both the brain and peripheries in the resting state), exclusively during NREM3 sleep. This intrinsic brain activity may alter the global fMRI signal in NREM3 sleep, causing it to become uncoupled from peripheral signals. Although the exact functions and/or mechanisms of action of these oscillations remain unclear, observations from the current study and a few prior studies could be thought of as a direct consequence of their action. For example, these intrinsic oscillations might be responsible for NREM3 sleep's hypothesised function of restoring hemodynamics (Javaheri and Redline 2012). Mounting evidence supports the link between the duration of NREM3 sleep and risk for vascular disorders. A decrease in the duration of slow wave sleep has been shown to increase the risk of developing hypertension (Fung et al. 2011) and stroke (Bassetti and Aldrich 2001; Carvalho et al. 2023). Moreover, wake‐up strokes were found to occur very rarely during slow wave sleep (Pérez‐Carbonell and Bashir 2020), attributed to its significantly lower autonomic variability. Our data also found that the contribution from autonomic activity is significantly lowered in brain LFOs during NREM3 (Figure S2), which might be a direct result of this active intrinsic brain mechanism aimed at reducing cerebrovascular tone in an attempt to maintain/restore optimal cerebrovascular activities. In addition to non‐neuronal systemic LFOs, neural activity also represents a potential source of cerebrovascular oscillations. Interestingly, we found that consistent neural activity peaks were absent prior to LF cerebrovascular changes (See Figure S3), indicating that there might be an active intrinsic brain mechanism associated with NREM3 sleep state that works to reduce the cerebral vasomotor activities for maintenance/restoration of optimal hemodynamic functions. Additionally, NREM3 sleep state‐related regional differences in cerebral perfusion may also contribute to the observed decoupling between global brain and peripheral hemodynamics.

Finally, it may be noted that the delays between cerebral and peripheral LFOs show high variability between participants and states (Figure 4B). This aligns with past research and may be explained by individual differences in length, diameter, and elasticity of the distinct blood vessel routes through which the LFOs travel from their source to the destination (i.e., brain or fingertip). In addition, differences in height, age, and gender of the participants could also play a role in this (Tong et al. 2012). Additionally, the delays tend to become negative (i.e., LFOs first appear in the brain) during light sleep (NREM1 and NREM2) compared to wakefulness. This may be reflective of the increased amplitudes of cerebrovascular oscillations in NREM1 and NREM2 (Figure 2), since regional circulatory changes, including local oxygen demands and vasodilation, could also affect the delays between these signals (Guyton and Hall 2011).

### 4.3 Limitations and Conclusions

Limitations of the current study include a lower sample size and a lack of recordings from additional peripheral locations such as the ear lobe, which, unlike the fingertips, has both sympathetic and parasympathetic innervation. A comparison between different peripheral locations and the brain would have helped clarify the state‐related regional haemodynamic changes better. In addition, this study also lacks data during REM sleep with a higher autonomic variability than NREM, which is essential for a comprehensive analysis. In conclusion, the current study shows that haemodynamics in the brain distinctly differ in both oscillation amplitude and spectral power from peripheral haemodynamics during NREM sleep. More importantly, the LF coupling between cerebral and peripheral haemodynamics becomes desynchronized during NREM3 sleep, suggestive of its vascular restorative function.

## Author Contributions

**Vidhya V. Nair:** writing – original draft, writing – review and editing, methodology, software, formal analysis, conceptualization, project administration. **Brianna R. Kish:** writing – review and editing, project administration. **Hideyuki Oshima:** formal analysis. **Qiuting Wen:** writing – review and editing. **Yunjie Tong:** conceptualization, methodology, software, formal analysis, writing – review and editing, funding acquisition, supervision. **A. J. Schwichtenberg:** conceptualization, writing – review and editing, formal analysis, supervision, methodology, software, funding acquisition.

## Conflicts of Interest

The authors declare no conflicts of interest.

## Supporting information


**Table S1:** Durations (in minutes) of sleep–wake states for each participant used in the analysis. Since participant #3 had a comparatively larger duration of NREM3, the group‐level results were validated by repeating all statistical analyses after excluding data from this participant.
**Figure S2:** Average power variations in the specific LFO range of 0.02–0.04 Hz in GS and NIRS signals from wake to NREM3. GS—global signal; NIRS—near‐infrared spectroscopy; LFO—low frequency oscillations; NREM—non rapid eye movement sleep; * (*p*‐value < 0.05).
**Figure S3:** A LF Cerebral hemodynamic change peak‐locked folding average analysis of EEG signals in the slow wave activity (SWA) range was performed to expose neural SWA, if any, prior to LF cerebral hemodynamic changes in both directions from wake to NREM3 states. The analysis method is described in detail in our previous study (Vijayakrishnan Nair et al. 2023). In short, the amplitude envelope of the EEG signal (Cz), filtered in the range of 0.2–4 Hz and smoothed with a moving average filter of 4 s, prior to each of the identified local maxima and minima (for outflow and inflow directions respectively) in the d/dt(LFO_GS_) signal was averaged for 6 s. A similar analysis was also performed during wakefulness by using the full‐spectrum EEG signals (bandpass‐filtered in the range of 0.1–45 Hz) from the same electrode. The results show that neural SWA occurs (~2–4 s) before cerebral hemodynamic changes in both directions only during light NREM sleep states. The mean signal in each case is illustrated with thick black and red lines, with standard deviation across participants represented by grey and orange regions around the mean signal. A.U—arbitrary units; EEG—electroencephalogram; GS—global signal; NIRS—near‐infrared spectroscopy; LFO—low frequency oscillations; NREM—non rapid eye movement sleep.

## Data Availability

The data that support the findings of this study are available on request from the corresponding author. The data are not publicly available due to privacy or ethical restrictions.
