# Supplementary material for: Cerebral and Peripheral Hemodynamics Across Wakefulness and NREM Sleep
Source: J Sleep Res. 2025 Aug 20;35(2):e70180. doi: 10.1111/jsr.70180 (PMC13003264; doi:10.1111/jsr.70180)
Supplement: Supplementary file 1 — Table S1: Durations (in minutes) of sleep–wake states for each participant used in the analysis. Since participant #3 had a comparatively larger duration of NREM3, the group‐level results were validated by repeating all statistical analyses after excluding data from this participant. Figure S2: Average power variations in the specific LFO range of 0.02–0.04 Hz in GS and NIRS signals from wake to NREM3. GS—global signal; NIRS—near‐infrared spectroscopy; LFO—low frequency oscillations; NREM—non rapid eye movement sleep; * (p‐value < 0.05). Figure S3: A LF Cerebral hemodynamic change peak‐locked folding average analysis of EEG signals in the slow wave activity (SWA) range was performed to expose neural SWA, if any, prior to LF cerebral hemodynamic changes in both directions from wake to NREM3 states. The analysis method is described in detail in our previous study (Vijayakrishnan Nair et al. 2023). In short, the amplitude envelope of the EEG signal (Cz), filtered in the range of 0.2–4 Hz and smoothed with a moving average filter of 4 s, prior to each of the identified local maxima and minima (for outflow and inflow directions respectively) in the d/dt(LFOGS) signal was averaged for 6 s. A similar analysis was also performed during wakefulness by using the full‐spectrum EEG signals (bandpass‐filtered in the range of 0.1–45 Hz) from the same electrode. The results show that neural SWA occurs (~2–4 s) before cerebral hemodynamic changes in both directions only during light NREM sleep states. The mean signal in each case is illustrated with thick black and red lines, with standard deviation across participants represented by grey and orange regions around the mean signal. A.U—arbitrary units; EEG—electroencephalogram; GS—global signal; NIRS—near‐infrared spectroscopy; LFO—low frequency oscillations; NREM—non rapid eye movement sleep. [file JSR-35-e70180-s001.docx]

***Supplemental Data***

***S1. Duration of sleep-wake states across participants***

*Table S1: Durations (in minutes) of sleep-wake states for each participant used in the analysis. Since participant #3 had a comparatively larger duration of NREM3, the group-level results were validated by repeating all statistical analyses after excluding data from this participant.*

***S2. Spectral power in the LFO band of autonomic neural origin significantly increases in the brain during light NREM sleep***

*Figure S2: Average power variations in the specific LFO range of 0.02- 0.04 Hz in GS and NIRS signals from wake to NREM3. GS – Global Signal; NIRS – Near-Infrared Spectroscopy; LFO – Low frequency Oscillations; NREM – Non Rapid Eye Movement sleep; * (p-value < 0.05).*

***S3. Neural SWA before CBV changes from wake to NREM3***

*Figure S3: A LF Cerebral hemodynamic change peak-locked folding average analysis of EEG signals in the slow wave activity (SWA) range was performed to expose neural SWA, if any, prior to LF cerebral hemodynamic changes in both directions from wake to NREM3 states. The analysis method is described in detail in our previous study*(Vijayakrishnan Nair et al., 2023)*. In short, the amplitude envelope of the EEG signal (Cz), filtered in the range of 0.2 – 4 Hz and smoothed with a moving average filter of 4 seconds, prior to each of the identified local maxima and minima (for outflow and inflow directions respectively) in the d/dt(LFO_GS_) signal was averaged for 6 seconds. A similar analysis was also performed during wakefulness by using the full-spectrum EEG signals (bandpass-filtered in the range of 0.1 Hz – 45 Hz) from the same electrode. The results show that neural SWA occurs (~ 2 – 4 seconds) before cerebral hemodynamic changes in both directions only during light NREM sleep states. The mean signal in each case is illustrated with thick black and red lines, with standard deviation across participants represented by gray and orange regions around the mean signal. A.U – Arbitrary Units; EEG – Electroencephalogram; GS – Global Signal; NIRS – Near-Infrared Spectroscopy;* *LFO – Low frequency Oscillations; NREM – Non Rapid Eye Movement sleep.*
